# Incidence of acute otitis media in children below 6 years of age seen in medical practices in five East European countries

**DOI:** 10.1186/s12887-016-0638-2

**Published:** 2016-07-26

**Authors:** Vytautas Usonis, Teresa Jackowska, Sigita Petraitiene, Alicja Sapala, Andrea Neculau, Izabella Stryjewska, Raghavendra Devadiga, Monica Tafalla, Katsiaryna Holl

**Affiliations:** 1 Faculty of Medicine, Clinic of Children Diseases, Vilnius University, Santariskiu 4, LT-08406 Vilnius, Lithuania; 2 Department of Pediatrics, The Centre of Postgraduate Medical Education, Warsaw, Poland; 3 Department of Pediatrics, Bielanski Hospital, Warsaw, Poland; 4 Faculty of Medicine, Department of Fundamental and Prophylactic Sciences, University Transilvania Brasov, Brasov, Romania; 5 GSK Vaccines, Warsaw, Poland; 6 GSK Pharmaceuticals Ltd, Bangalore, India; 7 GSK Vaccines, Wavre, Belgium

**Keywords:** Acute otitis media, Children, Eastern Europe

## Abstract

**Background:**

Although acute otitis media (AOM) remains a major public health problem worldwide and brings economic burden on health care system and caregivers, little information is available about its epidemiology in Eastern Europe.

**Methods:**

We conducted an epidemiological, prospective, observational, multi-centre cohort study (NCT01365390) in five East European countries (Estonia, Lithuania, Poland, Romania and Slovenia) between June 2011 and January 2013 to determine the incidence and clinical characteristics of AOM among children aged < 6 years during 1 year.

**Results:**

AOM incidence was 160.7 cases (95 % confidence interval [CI]: 144.7–177.9) per 1000 person-years (PY) being the lowest in the < 1 year age group (92.3 cases [95 % CI: 59.7–136.2] per 1000 PY) and the highest in the 3– < 4 years age group (208.9 cases [95 % CI: 165.1–260.7] per 1000 PY). AOM incidence was similar across the countries, with the exception of Slovenia (340.3 cases [95 % CI: 278.3–412.0] per 1000 PY). There was a lower risk in breastfed children and a higher risk in those attending school/childcare or with allergies. AOM required 521 visits to the doctor. Antibiotics were prescribed for 276 (74.8 %) episodes with the lowest prescription rate in Estonia (51.4 %) and the highest in Romania (83.7 %). Complications were rare and hospitalisations occurred in 2 % of the cases.

**Conclusions:**

The disease burden of AOM in Eastern Europe is relevant and public health initiatives to reduce it should be considered.

**Trial registration:**

ClinicalTrial.gov NCT01365390.

## Background

Acute otitis media (AOM) remains a major public health problem worldwide with 80 % of children experiencing an episode before the age of 3 years [1]. AOM may have non-specific symptoms, have frequent recurrences, often require several visits to the health care practitioner and is a primary reason for antibiotic prescription in children [2, 3]. All of this brings challenges and economic burden to the everyday health care system [4, 5]. In addition to this, AOM brings emotional and economic burden to the caregivers and may have a negative impact on their quality of life [6].

Little information is available about specific epidemiology of AOM in Eastern Europe. One publication from Poland reported that at least 65 % of children suffer from AOM by the age of 2 years [7]. In Romania, the microbiology and antibiotic susceptibility was described but no data on incidence or clinical practice exists till date [8]. We therefore undertook this study to estimate the incidence and describe demographic and clinical characteristics of AOM in children less than 6 years of age visiting Primary Care Paediatricians in five East European countries (Estonia, Lithuania, Poland, Romania and Slovenia).

## Methods

This prospective, observational, multi-centre cohort study (NCT01365390) was conducted at 29 primary healthcare clinics in five East European countries (Estonia = 1, Lithuania = 3, Poland = 10, Romania = 5 and Slovenia = 10) between 10 June 2011 and 20 January 2013. In addition, retrospective data from the previous year or from birth for children younger than 1 year of age at enrolment was collected for all children.

Children were selected from the paediatric population registered at the participating practices and parents were contacted via mail, telephone or during a routine visit. Children with an AOM episode and/or upper respiratory tract infection at the time of enrolment were excluded from participating in the study; children with no available medical history from the previous year or from birth if less than 1 year of age at enrolment were also excluded. Written informed consent was obtained from parents of all participating children before any study procedure was performed.

Of the 377,750 children who were registered in the practices, 2258 were enrolled in our study. One was excluded due to protocol violation and 2253 were valid for the follow-up analysis.

Demographics as well as information on pneumococcal vaccination history, method of feeding, day-care/school attendance, number of household siblings, prematurity, allergies, and exposure to indoor cigarette smoke were collected (Table 1). Medical records of the enrolled children were reviewed to retrieve information on physician-diagnosed AOM episodes during the year before enrolment or since birth for children younger than 1 year of age.

**Table 1:** Demographic characteristics of enrolled children

Medical record review^a^							
Characteristics		Estonia	Lithuania	Poland	Romania	Slovenia	Total
	*N*	250	300	1106	301	300	2257
Age (months)	Median (range)	36 (3–71)	27.5 (0–71)	24 (0–71)	34 (2–71)	27 (1–70)	28 (0–71)
Gender, n (%)	Female	122 (48.8)	154 (51.3)	554 (50.1)	139 (46.2)	157 (52.3)	1126 (49.9)
	Male	128 (51.2)	146 (48.7)	552 (49.9)	162 (53.8)	143 (47.7)	1131 (50.1)
Pneumococcal vaccination (at least 1 dose), n (%)	Yes	21 (8.4)	35 (11.7)	513 (46.4)	47 (15.6)	45 (15.0)	661 (29.3)
	No	227 (90.8)	265 (88.3)	590 (53.3)	254 (84.4)	255 (85.0)	1591 (70.5)
	Unknown	2 (0.8)	0 (−)	3 (0.3)	0 (−)	0 (−)	5 (0.2)
Child feeding, n (%)	Breast-fed	14 (5.6)	62 (20.7)	193 (17.5)	26 (8.6)	32 (10.7)	327 (14.5)
	Formula-fed	2 (0.8)	32 (10.7)	164 (14.8)	39 (13.0)	25 (8.3)	262 (11.6)
	Regular food	243 (97.2)	245 (81.7)	837 (75.7)	259 (86.0)	269 (89.7)	1853 (82.1)
Child care/school attendance, n (%)	2–5 children	25 (10.0)	1 (0.3)	32 (2.9)	100 (33.2)	12 (4.0)	170 (7.5)
	6–10 children	13 (5.2)	1 (0.3)	9 (0.8)	15 (5.0)	6 (2.0)	44 (1.9)
	> 10 children	175 (70.0)	145 (48.3)	350 (31.6)	144 (47.8)	185 (61.7)	999 (44.3)
Follow-up period^b^							
Characteristics		Estonia	Lithuania	Poland	Romania	Slovenia	Total
	*N*	249	299	1104	301	300	2253
Age (months)	Median (range)	36 (3–71)	28 (0–71)	24 (0–71)	34 (2–71)	27 (1–70)	28 (0–71)
Gender, n (%)	Female	121 (48.6)	153 (51.2)	553 (50.1)	139 (46.2)	157 (52.3)	1123 (49.8)
	Male	128 (51.4)	146 (48.8)	551 (49.9)	162 (53.8)	143 (47.7)	1130 (50.2)
	*N* ^c^	249	298	1100	300	298	2245
Pneumococcal vaccination (at least 1 dose), n (%)^d^	Yes	14 (5.6)	15 (5.0)	200 (18.2)	15 (5.0)	11 (3.7)	255 (11.4)
	No	235 (94.4)	283 (95.0)	884 (80.4)	285 (95.0)	287 (96.3)	1974 (87.9)
	Unknown	0 (−)	0 (−)	16 (1.5)	0 (−)	0 (−)	16 (0.7)

*N* total number of subjects

n (%) number (percentage) of subjects in each group

^a^Only 1 subject from Poland was excluded from the According-To-Protocol (ATP) cohort for retrospective analysis for protocol violation

^b^5 subjects (1 subject from Poland for protocol violation and 1 subject each from Estonia and Lithuania and 2 subjects from Poland for lost to follow-up) were excluded from the ATP cohort for prospective analysis

^c^Number of subjects available at study conclusion

^d^Sum of percentages may exceed 100 % due to rounding

Parents were instructed to visit the doctor each time the child had symptoms of AOM and/or upper respiratory tract infection within 12 months after enrolment. AOM was diagnosed by the investigator based on clinical judgement. Detailed information on the signs, symptoms, severity [using the Otoscopic Severity (OS)-8 scale for assessment of tympanic membrane and AOM Faces scale for the evaluation of severity of AOM symptoms] [9], course and treatment were recorded. No samples were collected as part of this study.

After 14–21 days of the first visit to an investigator for a new episode of AOM, the parents/guardians were contacted by telephone to collect information on the evolution and management of the episode.

Additionally, parents were contacted every 2 months to report any AOM symptoms and/or respiratory tract infection lasting ≥ 48 h, experienced by their child in the previous 2 months if not reported to the investigator. They were also required to report all visits made to other health care professionals (including all hospitalisations, emergency room visits, etc.) for any AOM or respiratory tract infection lasting ≥ 48 h.

Standard practices and recommendations in each country were followed for treatment and follow-up of each episode of AOM until resolution.

The study adhered to the Good Clinical Practice guidelines, including the Declaration of Helsinki and was conducted according to applicable regulatory requirements. The study was reviewed and approved by a national, regional, or investigational centre Independent Ethics Committee/Institutional Review Board.

The target sample size was 2200 children for an expected incidence of AOM of 26.8 cases per 100 person-years (PY) [10] (95 % Exact Poisson confidence interval (CI) [24.70–29.01]). The overall incidence of AOM episodes and the incidence by country were estimated with 95 % CI. The incidence of AOM was expressed as number of AOM cases per 1000 PY. The severity of AOM was described in terms of frequency of signs and symptoms, hospitalisations and complications. Crude and adjusted odds ratios (OR) and their 95 % CI were calculated using logistic regression analysis. The statistical analyses were performed using the Statistical Analysis System (*SAS*-version 9.2) Drug and Development (*SDD*) web portal version 3.5 and *Microsoft Excel* 2007.

## Results

The median age of the 2258 enrolled children was 28 months (range: 0–71 months), and 50.2 % (1130) were male; 14.5 % (327) of children were breast-fed and 53.7 % (1213) of children attended day-care service (Table 1).

At least one dose of pneumococcal conjugate vaccine (PCV) had been administered to 661 (29.3 %) children prior to enrolment, the lowest being in Estonia (21; 8.4 %) and the highest in Poland (513; 46.4 %) (Table 1). In addition, at least one dose of PCV was administered to 14 (5.6 %) children in Estonia, and 15 (5.0 %) each in Lithuania and Romania during the study follow-up period.

The overall incidence of physician-diagnosed AOM during the 1 year follow-up period was 160.7 cases (95 % CI: 144.7–177.9) per 1000 PY. The incidence was the lowest in Poland (115.7 [95 % CI: 96.7–137.3] cases per 1000 PY) and highest in Slovenia (340.3 [95 % CI: 278.3–412.0] cases per 1000 PY) (Table 2).

**Table 2:** Incidence of AOM episodes as diagnosed by a primary care physician or an otolaryngology specialist

	Medical record review		Follow-up period	
	Total number of AOM episodes (total population)	Incidence per 1000 person-years (95 % CI)	Total number of AOM episodes (total population)	Incidence per 1000 person-years (95 % CI)^a^
Estonia	24 (250)	97.7 (62.6–145.3)	35 (249)	137.8 (96.0–191.7)
Lithuania	58 (300)	225.2 (171.0–291.2)	55 (299)	184.0 (138.6–239.6)
Poland	121 (1106)	130.5 (108.3–155.9)	131 (1104)	115.7 (96.7–137.3)
Romania	34 (301)	118.7 (82.2–165.9)	43 (301)	141.9 (102.7–191.1)
Slovenia	125 (300)	455.3 (379.0–542.5)	105 (300)	340.3 (278.3–412.0)
Overall total	362 (2257)	181.8 (163.5–201.5)	369 (2253)	160.7 (144.7–177.9)

There is no overlap of dates between “Medical record review” and “Follow-up” period. The subjects were followed-up from June 2011 to January 2013

The medical records were reviewed 12 months prior to enrolment (entire birth period for subjects aged below 1 year)

*AOM* acute otitis media, *CI* confidence interval

^a^Based on the actual surveillance period since some subjects were followed-up for more than 1 year

The incidence of AOM was the lowest in the < 1 year age group (92.3 cases [95 % CI: 59.7–136.2] per 1000 PY) and the highest in the 3– < 4 years age group (208.9 cases [95 % CI: 165.1–260.7] per 1000 PY).

No difference was found when comparing vaccinated and non-vaccinated children: the incidence of AOM was 146.6 (95 % CI: 103.7–201.2) per 1000 PY in children who received at least one dose of PCV and 163.3 (95 % CI: 146.1–181.9) per 1000 PY in children who did not receive PCV.

**Fig. 1:**
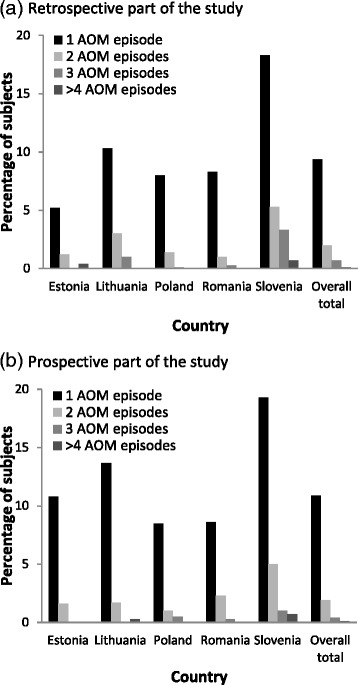
Percentage of subjects with 1, 2, 3, or 4 or more acute otitis media episodes as diagnosed by a physician or an otolaryngology specialist, by country during the retrospective (**a**) and prospective (**b**) parts of the study

A total of 246 (10.9 %) children experienced one AOM episode, 42 (1.9 %) two episodes and 12 (0.5 %) three or more during the follow-up study period (Fig. 1). Overall, 96 (26.0 %) of these episodes (Estonia: 1 [0.3 %]; Lithuania: 44 [11.9 %]; Poland: 16 [4.3 %]; Romania: 8 [2.2 %]; Slovenia: 27 [7.3 %]) required more than one visit to the doctor. The most frequently reported symptoms were ear pain/otalgia (271; 75.1 %) followed by fever (208; 57.6 %) (Table 3). Seven complications occurred after 369 AOM episodes (Table 3).

**Table 3:** Clinical and health economic characteristics of AOM episodes during the follow-up period

Characteristics	Estonia	Lithuania	Poland	Romania	Slovenia	Total
*Clinical signs*/*symptoms*						
*N* ^a^	34	52	131	43	101	361
Ear Pain/Otalgia, n (%)	30 (88.2)	40 (76.9)	101 (77.1)	33 (76.7)	67 (66.3)	271 (75.1)
Ear discharge, n (%)	4 (11.8)	2 (3.9)	4 (3.1)	1 (2.3)	7 (6.9)	18 (5.0)
Hearing loss, n (%)	11 (32.4)	6 (11.5)	3 (2.3)	0 (−)	4 (4.0)	24 (6.7)
Ear tugging, n (%)	5 (14.7)	1 (1.9)	4 (3.1)	26 (60.5)	15 (14.9)	51 (14.1)
Perforation of tympanic membrane, n (%)	1 (2.9)	0 (−)	2 (1.5)	0 (−)	5 (5.0)	8 (2.2)
Fever, n (%)	11 (32.3)	19 (36.5)	77 (58.8)	38 (88.4)	63 (62.4)	208 (57.6)
*Hospitalisation and complications*						
*N*	35	55	131	43	105	369
Child referred to hospital due to AOM, *n* (%)	0 (−)	2 (3.6)	7 (5.3)	0 (−)	1 (1.0)	10 (2.7)
Was the child hospitalised, n (% of those referred)	-	1 (50.0)	6 (85.7)	-	1 (100.0)	8 (80.0)
Complications, n (%)	3 (8.6)	0 (−)	3 (2.3)	1 (2.3)	0 (−)	7 (1.9)
*Procedures performed* ^b^						
*N*	1	0	36	0	3	40
Adenoidectomy	0 (−)	0 (−)	1 (2.8)	0 (−)	0 (−)	1 (2.5)
Ventilation tube insertion	1 (100.0)	0 (−)	0 (−)	0 (−)	0 (−)	1 (2.5)
Cleaning of tube	0 (−)	0 (−)	36 (100.0)	0 (−)	3 (100.0)	39 (97.5)
*Severity score* ^c^						
*N*	35	55	131	43	105	369
1, n (%)	5 (14.3)	2 (3.6)	4 (4.0)	1 (2.4)	5 (5.0)	17 (5.1)
2, n (%)	10 (28.6)	3 (5.5)	28 (27.7)	0 (−)	11 (10.9)	52 (15.6)
3, n (%)	5 (14.3)	10 (18.2)	34 (33.7)	5 (12.2)	11 (10.9)	65 (19.5)
4, n (%)	0 (−)	10 (18.2)	17 (16.8)	23 (56.1)	27 (26.7)	77 (23.1)
5, n (%)	5 (14.3)	11 (20.0)	10 (9.9)	9 (22.0)	24 (23.8)	59 (17.7)
6, n (%)	5 (14.3)	13 (23.6)	6 (5.9)	2 (4.9)	20 (19.8)	46 (13.8)
7, n (%)	5 (14.3)	6 (10.9)	2 (2.0)	1 (2.4)	3 (3.0)	17 (5.1)
Missing confirmed, n (%)	0 (−)	0 (−)	30 (−)	2 (−)	4 (−)	36 (−)
*Caregiver missed work* ^c^						
*N* ^d^	16	54	121	43	101	335
Yes	5 (31.3)	11 (20.4)	30 (24.8)	2 (4.7)	28 (27.7)	76 (22.7)
No	11 (68.8)	43 (79.6)	91 (75.2)	41 (95.3)	73 (72.3)	259 (77.3)

*AOM* acute otitis media

*N* total number of AOM episodes

n (%) number (percentage) of AOM episodes in each group

^a^Total number of AOM episodes for which signs/symptoms were recorded during at least one of the visit

^b^Sum of percentages may exceed 100 %, since more than one procedure had been performed

^c^Sum of percentages may exceed 100 % due to rounding

^d^Total number of AOM episodes for which health economics questionnaire was available

Ten episodes (2.7 %) were referred to the hospital, of which 80.0 % required subsequent hospitalisation and most cases (77; 23.1 %) were of a severity rating of 4 on the AOM Faces Scale (Table 3).

Antibiotics have been prescribed for at least 276 (74.8 %) episodes in all countries with the lowest prescription rate of 51.4 % in Estonia and the highest rate of 83.7 % in Romania. Parents took days off work due to episodes of AOM in their children in 22.7 % of the cases with some country variation between 4.7 to 31.3 %.

When looking at the year before enrolment into the study, it was found that a total of 212 (9.4 %) children experienced one AOM episode, 46 (2.0 %) two episodes, 18 (0.8 %) three or more (Fig. 1). Overall, 78 (21.5 %) of these episodes (Estonia: 1 [0.3 %]; Lithuania: 40 [11.0 %]; Poland: 13 [3.6 %]; Romania: 1 [0.3 %]; Slovenia: 23 [6.4 %]) required more than one visit to the doctor due to AOM. Complications were recorded for three AOM episodes. Antibiotics were prescribed for at least 298 (82.5 %) AOM episodes in all countries with the lowest prescription rate of 73.5 % in Romania and the highest rate of 92.8 % in Slovenia. AOM episodes led to hospital referrals in 5.3 % cases, of which 89.5 % required subsequent hospitalisation. Tympanocentesis was performed for one episode (1.9 %).

Protective effect of breast-feeding against AOM has been revealed in univariate analysis (adjusted OR: 0.19; 95 % CI: 0.08−0.44). On the contrary, increased risk of AOM was found in the children attending child care or school (especially with higher number of children per child care) (adjusted OR: 1.92; 95 % CI: 1.35–2.75) and for those with allergies (adjusted OR: 1.64; 95 % CI: 1.09–2.45) (Table 4). There was no significant association between AOM and other risk factors such as age, gender, pneumococcal vaccination, presence of household siblings, exposure to smoking, and premature birth.

**Table 4:** Odds ratios (95 % confidence intervals) of AOM associated with different demographic and clinical characteristics of the child

Characteristics	Categories	Crude OR (95 % CI)	Adjusted OR (95 % CI)
Age [Months]	-	1.02 (1.01–1.03)	1.00 (0.99–1.01)
Gender	Male	Reference	Reference
	Female	1.02 (0.79–1.31)	1.02 (0.79–1.32)
Child receive at least one dose of a Pneumococcal vaccine	No	Reference	Reference
	Yes	0.98 (0.74–1.29)	1.04 (0.78–1.38)
Breast-fed	No	Reference	Reference
	Yes	0.12 (0.05–0.26)	0.19 (0.08–0.44)
Number of household siblings	0	Reference	Reference
	≥ 1	1.38 (1.06–1.81)	1.20 (0.91–1.58)
Child care/school attendance	No	Reference	Reference
	Yes	2.68 (2.03–3.54)	1.92 (1.35–2.75)
Premature (< 37 weeks gestation)	No	Reference	Reference
	Yes	0.73 (0.38–1.42)	0.76 (0.39–1.49)
Allergies	No	Reference	Reference
	Yes	1.80 (1.22–2.66)	1.64 (1.09–2.45)
Child regularly exposed to cigarettes smoke indoors	No	Reference	Reference
	Yes	1.04 (0.66–1.62)	1.02 (0.65–1.62)

*AOM* acute otitis media, *CI* confidence interval, *OR* odds ratio, *95 % CI of OR* 95 % Wald confidence interval of odds ratio

## Discussion

This study was conducted to determine the incidence of AOM in children less than 6 years of age in Estonia, Lithuania, Poland, Romania and Slovenia. We observed that a minimal variation in AOM incidence exists across the five East European countries. Although the incidence of AOM in this study (160.7 cases (95 % CI: 144.7–177.9) per 1000 PY) was lower than what was previously reported in a similar study conducted in Western Europe (256 cases [95 % CI: 243–270] per 1000 PY) [10], our findings emphasise the health burden due to AOM in children below 6 years of age in five East European countries (Estonia, Lithuania, Poland, Romania and Slovenia).

Indeed, the incidence of AOM in our study is also lower compared to what was reported in Finland (32 cases [95 % CI: 30–34] per 100 PY) [11], appears to be higher compared to that in the Czech Republic (83.3–125.2 cases per 1000 PY) [12] and similar to the incidence in Italy (168 cases [95 % CI: 167–169] per 1000 PY) [13]. This could be due to several factors such as seeking behaviour for treatment of AOM, diagnostic procedures [14, 15], socioeconomic status of the parents, climate and genetic predisposition [16].

Children in this study had on average 1.23 episodes, and each episode required 1.41 visits to the doctor. The number of the visits varied by country which could be due to specificity of health care practice in each country.

Antibiotic prescription for AOM in East European countries is confirmed to be relatively high with the exception of Estonia where this rate is lower and accounted for only 50 %. This could be due to well applied strategies on watch and see for the AOM symptoms.

Based on country experience in Lithuania, AOM is an important reason for antibiotic prescription, but practices could differ between countries. There also might be a tendency to access secondary care first which is an added reason for under-reported incidence of diseases like AOM.

At the time of this study, none of the participating countries had included PCV into their routine vaccination program.

The major symptom of AOM was ear pain/otalgia followed by ear discharge and hearing loss which was consistent with previous results [17].

As expected, breast feeding has a protective effect against AOM occurrence. This finding is in line with previously published studies [18–20]. In a review and meta-analysis article, it was observed that the infants fed with any formula during the first 6 months of life had twice the odds of developing AOM as breastfed children (OR: 2.00; 95 % CI: 1.40–2.78) [19]. In another study, it was seen that the percentage of children with AOM was lower among breast fed compared to formula fed; 2 % vs. 16 % in children aged 2 months (*p* = 0.01) and 13 % vs. 62 % in children aged 6 months (*p* = 0.0001) [20]. Therefore, as the World Health Organization recommends, breast feeding should occur at least for the first 6 months of infancy [21]. We also found an increased risk of AOM for children with allergies, as observed in another study [22]. The higher incidence of AOM in child attending care centres can be explained by the higher transmission rate observed there [23].

Parents required staying home and missing work due to AOM episode affecting their child in about 23 % of the cases, which can be due to the fact that very often other relatives, especially retired grandparents, help with this.

Strengths of our study include the close follow-up of the study children that made it possible to detect AOM episodes even if the parents failed to communicate proactively the suspicion of a positive case. This could have also led to a limitation if parents gave the children medication for a fever prior to the AOM visit, resulting in under-reporting of the level of fever. All five countries in the study used the same standardised protocol thus facilitating country-wise comparisons. One limitation of this study is the case definition for AOM: clinical judgement made by general practitioners might have not been accurate, but primary care setting is a better proxy to general population epidemiology. If specialised clinics were to be included, the better accuracy of diagnosis would have been accompanied with a low representativeness of the studied population. Furthermore, the burden of AOM can be valuably assessed based on the prevalence of doubtful cases, without relying exclusively on the diagnosis of otolaryngology specialist [10]. Another limitation of the study is that no information were collected about those children not willing to participate or whose contact was not possible, so that we cannot ensure that they might not be different from those enrolled. Finally, there might be differences in case definitions of AOM between the countries under study.

## Conclusions

We conclude that the disease burden of AOM in Eastern Europe is relevant and consistent between the five countries. Public health initiatives, such as prevention through vaccination, should be considered to reduce the disease burden of AOM on the society and public health sector.

## Abbreviations

AOM, acute otitis media; CI, confidence interval; OR, odds ratio; OS, otoscopic severity; PCV, pneumococcal conjugate vaccine; PY, person-years; SAS, statistical analysis system; SDD, SAS drug and development
